# Low‐dose ionizing radiation‐induced RET/PTC1 rearrangement via the non‐homologous end joining pathway to drive thyroid cancer

**DOI:** 10.1002/mco2.690

**Published:** 2024-08-12

**Authors:** Yuhao Liu, Jiaojiao Zhu, Shenghui Zhou, Yifan Hou, Ziyan Yan, Xingkun Ao, Ping Wang, Lin Zhou, Huixi Chen, Xinxin Liang, Hua Guan, Shanshan Gao, Dafei Xie, Yongqing Gu, Ping‐Kun Zhou

**Affiliations:** ^1^ Beijing Key Laboratory for Radiobiology Beijing Institute of Radiation Medicine Beijing China; ^2^ Hengyang Medical College University of South China Hengyang China; ^3^ College of Life Sciences Hebei University Baoding China

**Keywords:** DNA‐PKcs, low‐dose ionizing radiation, NHEJ, RET/PTC1 rearrangement, thyroid cancer

## Abstract

Thyroid cancer incidence increases worldwide annually, primarily due to factors such as ionizing radiation (IR), iodine intake, and genetics. Papillary carcinoma of the thyroid (PTC) accounts for about 80% of thyroid cancer cases. RET/PTC1 (coiled‐coil domain containing 6 [CCDC6]‐rearranged during transfection) rearrangement is a distinctive feature in over 70% of thyroid cancers who exposed to low doses of IR in Chernobyl and Hiroshima‒Nagasaki atomic bombings. This study aims to elucidate mechanism between RET/PTC1 rearrangement and IR in PTC. N‐thy‐ori‐3‐1 cells were subjected to varying doses of IR (2/1/0.5/0.2/0.1/0.05 Gy) of IR at different days, and result showed low‐dose IR‐induced RET/PTC1 rearrangement in a dose‐dependent manner. RET/PTC1 has been observed to promote PTC both in vivo and in vitro. To delineate the role of different DNA repair pathways, SCR7, RI‐1, and Olaparib were employed to inhibit non‐homologous end joining (NHEJ), homologous recombination (HR), and microhomology‐mediated end joining (MMEJ), respectively. Notably, inhibiting NHEJ enhanced HR repair efficiency and reduced IR‐induced RET/PTC1 rearrangement. Conversely, inhibiting HR increased NHEJ repair efficiency and subsequent RET/PTC1 rearrangement. The MMEJ did not show a markable role in this progress. Additionally, inhibiting DNA‐dependent protein kinase catalytic subunit (DNA‐PKcs) decreased the efficiency of NHEJ and thus reduced IR‐induced RET/PTC1 rearrangement. To conclude, the data suggest that NHEJ, rather than HR or MMEJ, is the critical cause of IR‐induced RET/PTC1 rearrangement. Targeting DNA‐PKcs to inhibit the NHEJ has emerged as a promising therapeutic strategy for addressing IR‐induced RET/PTC1 rearrangement in PTC.

## Graphical abstract

We found that low‐dose IR can induce RET/PTC1 rearrangement in N‐thy‐ori‐3‐1 cell and there is a dose‐dependent manner between dose and rearrangement rate. We showed that this progress of IR‐induced RET/PTC1 rearrangement requires NHEJ and not HR. Moreover, DNA‐PKcs inhibition decreased the repair efficiency of NHEJ. Importantly, the RET/PTC1 rearrangement induced by IR was significantly decreased by DNA‐PKcs.

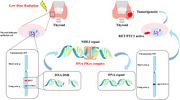

## 1 INTRODUCTION

Thyroid cancer is the most common endocrine cancer, and its incidence increases worldwide every year. The causes of thyroid cancer include iodine intake, genetics, and ionizing radiation (IR).^1^, ^2^, ^3^ Papillary carcinoma of the thyroid (PTC) accounts for about 80% of thyroid cancer cases. Numerous studies have demonstrated that the thyroid is sensitive to IR^3^; especially, exposure to IR during childhood or adolescence increases the risk of PTC.^4^, ^5^ Besides, among the survivors of Chernobyl and Hiroshima–Nagasaki atomic bombings, a significant increase in thyroid cancer incidence in adulthood was found after exposure to low doses of IR during adolescence and over 70% of thyroid cancers were accompanied by a distinctive feature: RET/PTC1 rearrangements.^6^, ^7^, ^8^ RET/PTC rearrangement is a prevalent genetic alteration observed in PTC and results in the oncogenic rearrangement of the RET gene. It is estimated that this fusion gene accounts for approximately 20%−40% of adult sporadic PTC cases.^9^, ^10^, ^11^ RET/PTC1 is a fusion protein formed by the fusion of the tyrosine kinase domain of the RET proto‐oncogene, which is situated on human chromosome 10, with the H4 gene (also known as CCDC6), located at 10q21 on the same chromosome. Chromosomal rearrangement always results from DNA recombination, repair, and so on.^12^, ^13^ RET/PTC rearrangements were observed in immortalized human thyroid cells (HTori‐3) after gamma radiation exposure.^14^ The detailed mechanism between RET/PTC1 rearrangement and DNA damage repair has not been reported.

Generally, rearrangement arises from the DNA damage repair after a double‐strand break (DSB). DNA DSB always resulted in reactive oxygen species from IR, oxidative metabolism, and chemical agents. The mechanisms of chromosomal rearrangement, including canonical non‐homologous end joining (NHEJ) and microhomology‐mediated end joining (MMEJ), have been well discussed.^15^, ^16^, ^17^, ^18^, ^19^ NHEJ has been identified that can form different rearrangements, such as translocations, inversions, and deletions.^12^, ^16^, ^20^ Whether NHEJ could participate in the RET/PTC rearrangement is not very clear. DNA‐dependent protein kinase catalytic subunit (DNA‐PKcs) is a serine/threonine protein kinase that is a critical regulator of NHEJ, which is involved in DNA damage repair. It has been widely discussed in DNA damage repair,^21^ tumors, and natural immunity.^22^, ^23^ The RET chromosomal rearrangement induced by low dose of IR has not been reported to be associated with DNA‐PKcs.

This study aimed to investigate the function and mechanism of low‐dose IR‐induced RET/PTC1 rearrangement in PTC. We performed nested PCR on normal human thyroid follicular epithelial cell line (N‐thy‐ori‐3‐1) treated with different low dose of IR to determine the RET/PTC1 rearrangement corresponding to low dose of IR and performed in vitro and in vivo functional validation to identify the function of RET/PTC1 rearrangement to PTC. Mechanistically, we investigated the key role of NHEJ in RET/PTC1 rearrangement and the specific regulatory relationships between NHEJ and DNA‐PKcs in normal human thyroid follicular epithelial cells exposed to low‐dose IR.

## 2 RESULTS

### 2.1 IR induces RET/PTC1 rearrangement in thyroid follicular epithelial cells in a dose‐dependent manner

It could be detected when RET/PTC1 rearrangement occurs in one cell and is mixed in 10^6^ cells (Figure S2A,B). RET/PTC1 rearrangement was not observed in either 0.2 or 0.5 Gy after 3 days of exposure to IR (Figure S2C). Three RET/PTC1 rearrangement‐positive wells were detected in the 0.2 Gy group, six RET/PTC1 rearrangement‐positive wells were detected in the 0.5 Gy group at the sixth day (Figure S2C), and 6 days was determined as the collection time in the following experiments.

**FIGURE 1:**
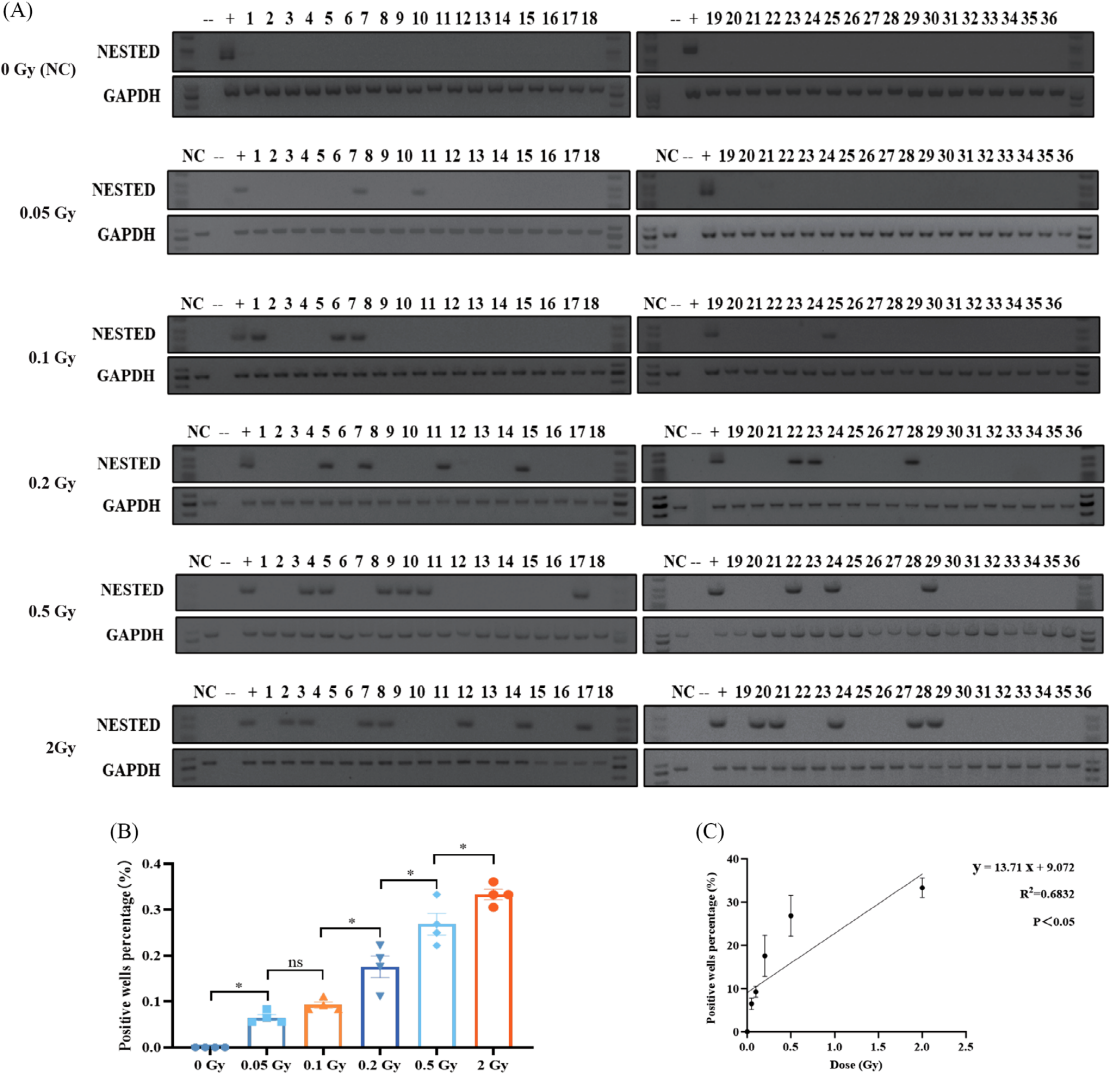
Ionizing radiation (IR) induces RET/PTC1 rearrangement in thyroid follicular epithelial cells in a dose‐dependent manner. (A) Detection of RET/PTC1 in non‐IR N‐thy‐ori‐3‐1 cells (control group) and detection of RET/PTC1 in Nthy‐ori‐3‐1 cells at 6 days with different doses: 0.05, 0.1, 0.2, 0.5, and 1 Gy. (B) The quantitation of (A). (C) Linear regression was performed on the dose and frequency of rearrangement. TPC1 cells were used as positive controls, negative controls done during the different steps without cDNA; GAPDH was used for normalization. Lane numbers of 1–36 correspond with independent cell wells. Data were derived from at least three independent experiments. The data are shown as means ± SEM. Statistical significance was calculated with the one‐way analysis of variance (ANOVA), ^*^ *p *< 0.05.

To further identify the association between the dose of IR and the number of RET/PTC1 rearrangement‐positive wells in normal human thyroid epithelial cells, N‐thy‐ori‐3‐1 cells were exposed to five doses (2/1/0.5/0.2/0.1/0.05 Gy), and detected the RET/PTC1 rearrangement at sixth day after IR (Figure 1A and Table 1). The result showed that the RET/PTC1 rearrangement increased after IR in a dose‐dependent manner (Figure 1B,C). Therefore, IR‐induced RET/PTC1 rearrangement might be a potential mechanism of IR‐induced PTC.

**TABLE 1:** Three independent experiments of detecting RET/PTC1 with different doses of 0 Gy (control), 0.05 Gy, 0.1 Gy, 0.2 Gy, 0.5 Gy, and 2 Gy.

	Treatments	Wells tested	Wells RET/PTC1 positives	RET/PTC1 (%)
Experiment 1	Control	36	0	0
	0.05 Gy	36	3	8.3
	0.1 Gy	36	4	11.1
	0.2 Gy	36	7	19.4
	0.5 Gy	36	9	25
	2 Gy	36	12	33.3
Experiment 2	Control	36	0	0
	0.05 Gy	36	3	8.3
	0.1 Gy	36	3	8.3
	0.2 Gy	36	4	11.1
	0.5 Gy	36	8	22.2
	2 Gy	36	11	30.6
Experiment 3	Control	36	0	0
	0.05 Gy	36	2	5.6
	0.1 Gy	36	3	8.3
	0.2 Gy	36	8	22
	0.5 Gy	36	9	25
	2 Gy	36	13	36.1

### 2.2 RET/PTC1 rearrangement promotes PTC tumorigenesis and progression in vitro and in vivo

**FIGURE 2:**
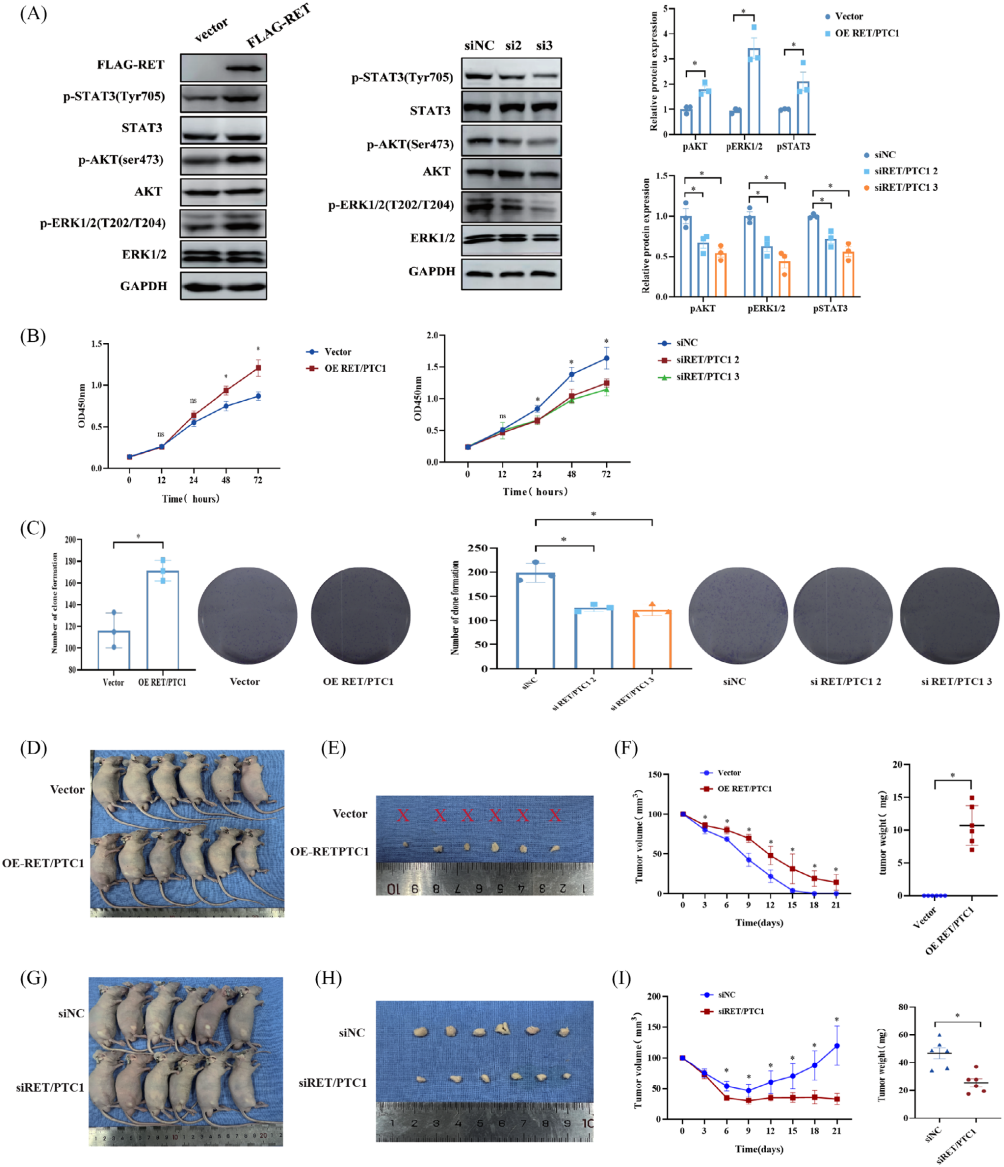
RET/PTC1 rearrangement promotes papillary carcinoma of the thyroid (PTC) tumorigenesis and progression in vitro and in vivo. (A) The expression of target protein downstream of RET was detected after RET/PTC1 overexpression or knockdown in N‐thy‐ori‐3‐1 and TPC‐1 cells. Statistical analysis of the intensity values of expression of the protein normalized to that of GAPDH. (B) Cell counting assays (CCK‐8) assays of N‐thy‐ori‐3‐1 and TPC‐1 cells after RET/PTC1 overexpression or knockdown. (C) Colony formation assays of N‐thy‐ori‐3‐1 and TPC‐1 cells after RET/PTC1 overexpression or knockdown. (D) Subcutaneous injection (s.c.) tumor model showed that N‐thy‐ori‐3‐1 cells were grew faster after RET/PTC1 overexpressed (*n* = 6). (E) The tumor volume from (D). (F) The tumor weight from (D). (G) s.c. tumor model showed while TPC‐1 cells significantly restricted after RET/PTC1 knockdown. (H) The tumor volume from (G). (I) The tumor weight from (G). The data are derived from at least three independent experiments and shown as means ± SEM. Comparison by unpaired *t*‐test, ^*^ *p *< 0.05.

We further assessed function of RET/PTC1 rearrangement in PTC and discovered that over‐expression of RET/PTC1 in the N‐thy‐ori‐3‐1 cells could activate AKT, ERK, and STAT3, all of which have been shown to be oncogenesis key signals that can be activated by RET/PTC1 (Figures 2A and S3A). In contrast, the inhibition of RET/PTC1 in the TPC‐1 cells could inactivate these oncogenesis signals (Figure 2A and S3B). In vitro, over‐expression of RET/PTC1 in N‐thy‐ori 3‐1 cells markedly increased the cell proliferation, as determined via cell counting assays (CCK‐8) and plate colony formation assays (Figure 2B,C). However, the CCK‐8 was used to identify that RET/PTC1 deletion could reduce the cell proliferation (Figure 2B). Concordantly, RET/PTC1 depletion also decreased the number of cell colonies, as determined by plate colony formation assays (Figure 2C). Furthermore, we verified the oncogenicity of RET/PTC1 in vivo. We observed that over‐expression of RET/PTC1 in N‐thy‐ori 3‐1 cells leads to a significant increase in the growth of subcutaneous xenograft tumors in nude mice (Figure 2D), including the sizes and weights of tumors (Figure 2E,F). In addition, knockdown of RET/PTC1 in TPC‐1 cells leads to a significant reduction in the growth of subcutaneous xenograft tumors in nude mice (Figure 2H,I). These results indicated that RET/PTC1 drives the formation and progression of PTC in vitro and in vivo.

### 2.3 NHEJ repair contributes to RET/PTC1 rearrangement induced by IR in thyroid follicular epithelial cells

**FIGURE 3:**
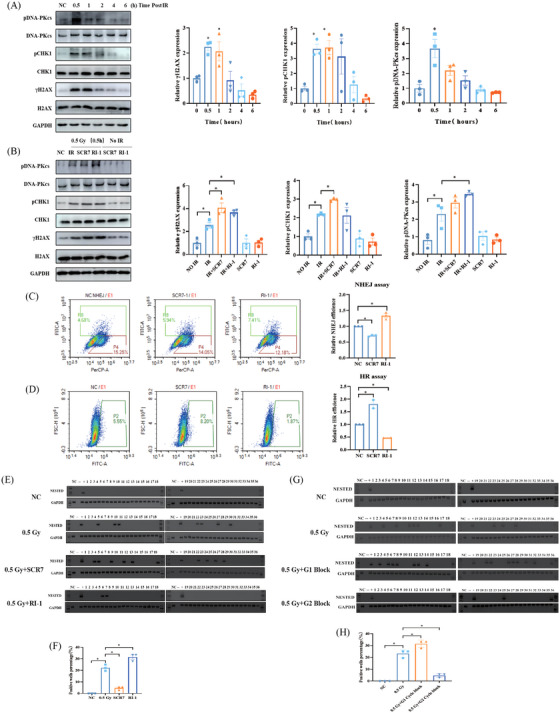
Non‐homologous end joining (NHEJ) repair contributes to RET/PTC1 rearrangement induced by ionizing radiation (IR) in thyroid follicular epithelial cells. (A) Western blot for detecting key proteins in DNA damage response (DDR) with 0.5 Gy after 0.5, 1, 2, 4, and 6 h. (B–D) N‐thy‐ori‐3‐1 cells treated with NHEJ inhibitor (SCR7) and homologous recombination (HR) inhibitor (RI‐1). (B) Detecting DDR proteins with 0.5 Gy. (C) NHEJ assay after treating with SCR7 or RI‐1. (D) HR assay after treating with SCR7 or RI‐1. (E) Detection of RET/PTC1 in N‐thy‐ori‐3‐1 cells at 6 days with different treatments: non‐irradiation N‐thy‐ori‐3‐1 cells (NC group), 0.5 Gy, 0.5 Gy combined with SCR7, and 0.5 Gy combined with RI‐1. (F) The quantitation of (E). (G) Detection of RET/PTC1 in N‐thy‐ori‐3‐1 cells at 6 days with different treatments: non‐irradiation N‐thy‐ori‐3‐1 cells (NC group), 0.5 Gy, 0.5 Gy combined with N‐thy‐ori‐3‐1 cells blocked in G1 phase, and 0.5 Gy combined with N‐thy‐ori‐3‐1 cells blocked in G2 phase. (H) The quantitation of (G). Lane numbers of 1–36 correspond with independent cell wells. The data are derived from at least three independent experiments. The data are shown as means ± SEM. Comparison by unpaired *t*‐test, ^*^ *p *< 0.05.

To determine the underlying mechanism of RET/PTC1 rearrangement, we found that γ‐H2AX and pCHK1 were highly expressed at 0.5 h exposed with 0.5 Gy IR, indicating that DNA damage reached its maximum after 0.5 h of IR (Figure 3A). The NHEJ repair pathway inhibitor SCR7 and the homologous recombination (HR) repair pathway inhibitor RI‐1 were employed to target DNA ligase IV and Rad51, respectively, which are key proteins in both pathways. A more increase in γ‐H2AX expression was detected after 0.5 Gy of IR combining use of SCR7 and RI‐1 than IR, and pCHK1 showed a same increase after the use of NHEJ inhibitors (Figure 3B). NHEJ and HR repair efficiency experiments showed that RI‐1 decreased the HR repair efficiency and increased the NHEJ repair efficiency (Figure 3C). Conversely, SCR7 decreased the NHEJ repair efficiency and increased the HR repair efficiency (Figure 3D). These findings indicate that NHEJ inhibition promoted HR repair and that HR increased that's of NHEJ (Figure 3C,D). Next, we investigated whether the DNA damage repair efficiency can regulate the rate of RET/PTC1 arrangement. The results showed that the number of RET/PTC1 rearrangement‐positive wells significantly increased after HR inhibition and decreased after NHEJ inhibition (Figure 3E,F and Table 2). In addition to canonical NHEJ and HR, the MMEJ is also an important DNA damage repair mechanism. To ascertain the role of MMEJ on RET/PTC1 rearrangement induced by IR, the MMEJ inhibitor Olaparib was employed to treat N‐thy‐ori‐3‐1 cells. Compared with NC group, the NHEJ and HR repair efficiency showed no significant alterations after treated with Olaparib (Figure S4A). Furthermore, the positive rate of IR‐induced RET/PTC1 rearrangement remained unmodified upon Olaparib treatment (Figure S4B,C). Additionally, the combining use of SCR7 and RI‐1 could also reduce the formation of RET/PTC1 induced by IR, and this reduction extend is consistent with the use of SCR7 (Figure S4B,C and Table S1). These findings indicted that the NHEJ not HR or MMEJ is the cause of IR‐induced RET/PTC1 rearrangement.

**TABLE 2:** Three independent experiments of detecting RET/PTC1 with different treatments: non‐irradiation Nthy‐ori‐3‐1 cells (NC group), 0.5 Gy, 0.5 Gy combined with SCR7, and 0.5 Gy combined with RI‐1.

	Treatments	Wells tested	Wells RET/PTC1 positives	RET/PTC1 (%)
Experiment 1	Control	36	0	0
	0. 5 Gy	36	7	19.4
	0.5 Gy + SCR7	36	1	2.8
	0.5 Gy + RI‐1	36	10	27.8
Experiment 2	Control	36	0	0
	0.05 Gy	36	9	25
	0.5 Gy + SCR7	36	2	5.6
	0.5 Gy + RI‐1	36	12	33.3
Experiment 3	Control	36	0	0
	0.05 Gy	36	9	25
	0.5 Gy + SCR7	36	2	5.6
	0.5 Gy + RI‐1	36	12	33.3

To further elucidate the involvement of NHEJ in RET/PTC1 rearrangement induced by IR. Studies have shown that the cell cycle phase is associated with the selective of DNA damage repair mechanisms: cells in the G1 phase predominantly utilize the NHEJ pathway, whereas cells in the G2 phase are more inclined to employ the HR pathway.^24^, ^25^ Therefore, the N‐thy‐ori‐3‐1 cells were treated with thymidine to achieve cell synchronization, and the G1 and G2 phase cells were separately exposed to IR (Figure S5A–C). Results indicated a significant increase in RET/PTC1 rearrangement in G1 phase cells compared to the control IR group (Figure 3G,H). In contrast, a notable decrease in RET/PTC1 rearrangement was observed in G2 phase cells relative to the IR group (Figure 3G,H and Table 3). These findings indicate that the induction of RET/PTC1 rearrangement by IR is specific to the G1 phase, emphasizing the role of NHEJ in this process.

**TABLE 3:** Three independent experiments of detecting RET/PTC1 with different treatments: non‐irradiation Nthy‐ori‐3‐1 cells (NC group), 0.5 Gy, 0.5 Gy combined with G1 block, and 0.5 Gy combined with G2 block.

	Treatments	Wells tested	Wells RET/PTC1 positives	RET/PTC1 (%)
Experiment 1	Control	36	0	0
	0.5 Gy	36	9	25
	0.5 Gy + G1 block	36	15	41.7
	0.5 Gy + G2 block	36	3	8.3
Experiment 2	Control	36	0	0
	0. 5 Gy	36	9	25
	0.5 Gy + G1 block	36	12	33.3
	0.5 Gy + G2 block	36	1	2.8
Experiment 3	Control	36	0	0
	0. 5 Gy	36	10	27.8
	0.5 Gy + G1 block	36	15	41.7
	0.5 Gy + G2 block	36	1	2.8

### 2.4 DNA‐PKcs‐mediated NHEJ contributes to IR‐induced RET/PTC1 rearrangement in thyroid follicular epithelial cells

**FIGURE 4:**
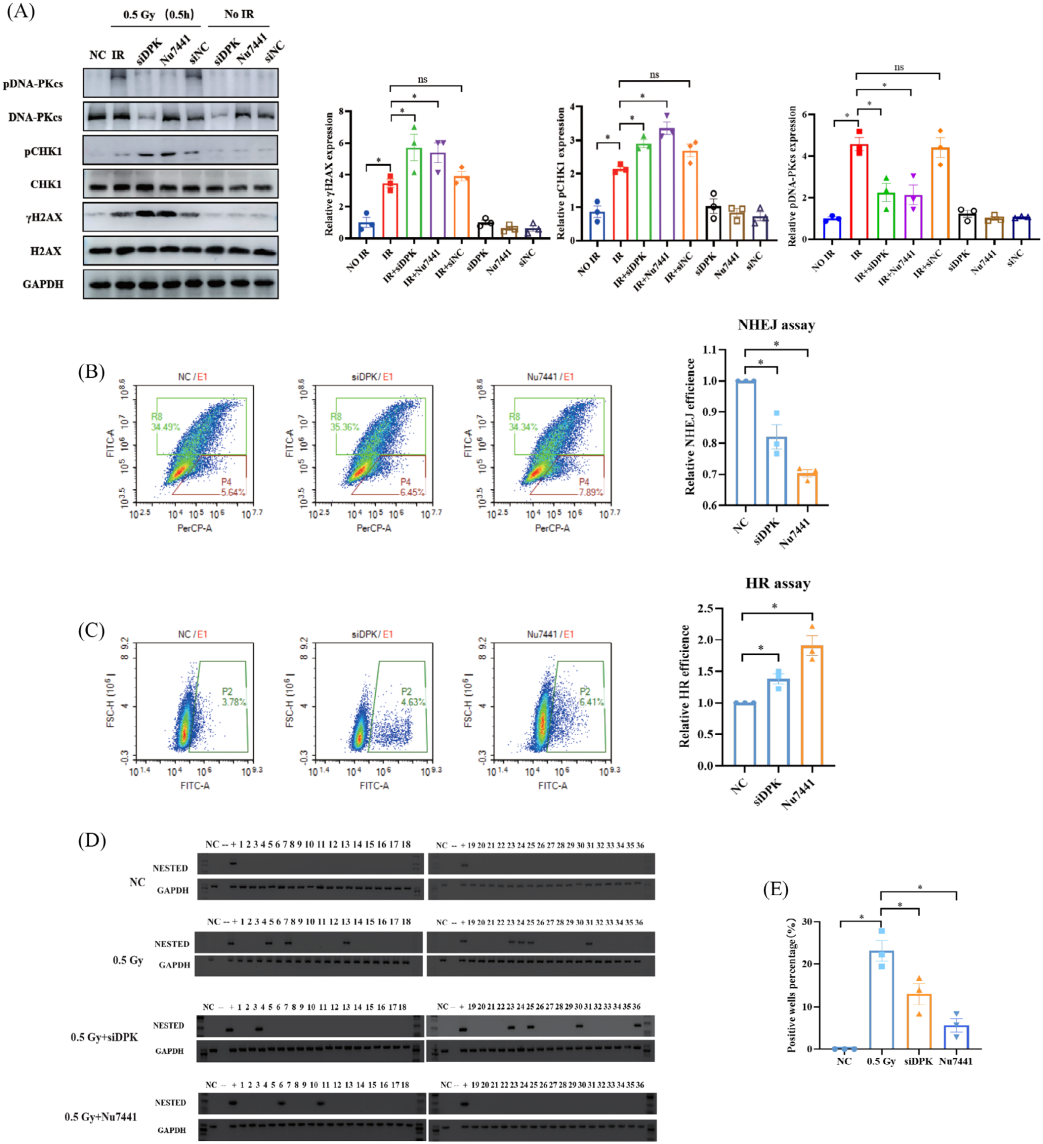
DNA‐dependent protein kinase catalytic subunit (DNA‐PKcs)‐mediated non‐homologous end joining (NHEJ) contributes to ionizing radiation (IR)‐induced RET/PTC1 rearrangement in thyroid follicular epithelial cells. (A) Inhibition of DNA‐PKcs by siDPK and Nu7441, Western blot for detecting key proteins in DDR after 0.5 Gy at 0.5 h. (B and C) N‐thy‐ori‐3‐1 cells treated with Nu7441 and siDPK to assay the NHEJ or homologous recombination (HR) repair efficiency. (B) NHEJ assay. (C) HR assay. (D) Detection of RET/PTC1 rearrangement in N‐thy‐ori‐3‐1 cells at 6 days with different treatments: non‐irradiation N‐thy‐ori‐3‐1 cells (NC group), 0.5 Gy, 0.5 Gy combined with siDPK, and 0.5 Gy combined with Nu7441. (E) The quantitation of (D). Lane numbers of 1–36 correspond with independent cell wells. Data are derived from at least three independent experiments. Data are shown as means ± SEM. Comparison by unpaired *t*‐test, ^*^ *p *< 0.05.

In view of the pivotal role of DNA‐PKcs in response to IR and NHEJ, we found that the pDNA‐PKcs was highly expressed at 0.5 Gy after 0.5 h (Figure 3A). Inhibition of DNA‐PKcs with siRNA‐DNA‐PKcs (siDPK) and kinase inhibitor Nu7441 could significantly promoted the IR‐induced pCHK1 expression increase (Figure 4A) and decrease the NHEJ repair efficiency (Figure 4A–C). To investigate the role of DNA‐PKcs in IR‐induced RET/PTC1 rearrangement generation, N‐thy‐ori‐3‐1 cells were subjected to simultaneous DNA‐PKcs inhibition and 0.5 Gy IR treatment using siDPK or Nu7441. The results showed a significant decrease in the number of RET/PTC1 rearrangement‐positive wells following DNA‐PKcs inhibition (Figure 4D,E and Table 4), suggesting that DNA‐PKcs‐mediated NHEJ was critical for the occurrence of RET/PTC1 rearrangements induced by IR. Taken together, these results demonstrated that DNA‐PKcs‐mediated NHEJ plays a crucial role in the occurrence of IR‐induced RET/PTC1 rearrangement.

**TABLE 4:** Three independent experiments of detecting RET/PTC1 with different treatments: non‐irradiation Nthy‐ori‐3‐1 cells (NC group), 0.5 Gy, 0.5 Gy combined with siDPK, and 0.5 Gy combined with Nu7441.

	Treatments	Wells tested	Wells RET/PTC1 positives	RET/PTC1 (%)
Experiment 1	Control	36	0	0
	0.5 Gy	36	7	19.4
	0.5 Gy + siDPK	36	5	13.9
	0.5 Gy + Nu7441	36	2	5.6
Experiment 2	Control	36	0	0
	0.05 Gy	36	8	22.2
	0.5 Gy + siDPK	36	3	8.3
	0.5 Gy + Nu7441	36	3	8.3
Experiment 3	Control	36	0	0
	0.05 Gy	36	10	27.8
	0.5 Gy + siDPK	36	6	16.7
	0.5 Gy + Nu7441	36	1	2.8

## 3 DISCUSSION

The escalating levels of global environmental pollutants, such as metal ions,^26^ air pollution,^27^ and endocrine‐disrupting chemicals,^28^ have been associated with a rise in thyroid cancer incidence both directly and indirectly. Thyroid cancer is a well‐known risk associated with exposure to IR,^14^ in addition to abnormal iodine consumption^29^ and family history.^30^ It has been observed that workers in the nuclear industry as well as doctors practicing nuclear medicine have also been exposed to such radiation following events such as Chernobyl and Fukushima.^31^ After the Chernobyl incident, there has been a significant surge in thyroid cancer occurrences, particularly PTC, which exhibited chromosomal rearrangements.^31^, ^32^ Conventional chromosomal rearrangements and the subsequent cellular consequences have been extensively recorded. However, the alarming rise in thyroid cancer incidence without a corresponding increase in mortality, may be attributed to the overdiagnosis of thyroid nodules detected by ultrasound.^33^, ^34^, ^35^ This issue of overdiagnosis has been observed in various geographical regions, including areas outside the contaminated zones following the Chernobyl accident, where there has been a heightened awareness and increased use of diagnostic imaging.

There are two types of chromosomal rearrangements in thyroid follicular epithelial cells caused by IR: RET/PTC1 and RET/PTC3.^14^ Previous reports indicate that RET/PTC1 occurs more frequently after exposing with IR.^36^ Nonetheless, the precise origins of thyroid cancer rearrangements remain unknown, with only the triggering conditions being outlined rather than the mechanism.^37^, ^38^, ^39^ Our findings revealed that even at low dose of IR (0.05 Gy) could induce RET/PTC1 rearrangement in normal thyroid follicular epithelial cells. And there is a dose‐dependent manner of IR induced RET/PTC1 rearrangement, which is consistent with previous study.^14^ Notably, previous studies have shown that RET/PTC1 rearrangement can be detected at 9^14^ or 14^40^ days after IR, whereas our study found that they can be detected at sixth day. This difference in detection time may be due to the limitation of the number of RET/PTC1 rearrangement‐positive cells. RET/PTC1 rearrangement has been confirmed in many PTC cases and thought to be a tumor driver gene.^41^, ^42^, ^43^ In this study, overexpression of RET/PTC1 induced the N‐thy‐ori‐3‐1 cells to have clonal and tumorigenic ability. We confirmed the carcinogenesis of RET/PTC1 rearrangement in PTC through both in vivo and in vitro experiments.

The RET/PTC1 rearrangement is a fusion gene resulting from RET and CCDC6. The fusion may occur due to a chromosome break caused by IR which ultimately leads to the formation of RET/PTC1.^44^, ^45^, ^46^ The potential regulatory mechanism responsible for this fusion gene in IR‐induced PTC has yet to be determined. In general, the fusion genes always form from two genes whose proximity is located at chromosome through DNA damage repair following a DNA DSB. DNA DSBs can be caused by both extrinsic and intrinsic factors. Extrinsic factors include sources such as IR and reactive oxygen species. Intrinsic factors include examples such as DNA replication errors and V(D)J recombination.^21^, ^47^, ^48^, ^49^ NHEJ is the main pathway for repairing of two‐ended DSBs and has been identified that can form different rearrangements such as translocations, inversions, and deletions.^50^, ^51^ In addition to the well‐established DNA damage repair pathways, such as HR and NHEJ, there are also non‐canonical end joining pathways, such as MMEJ, which might be a potentially regulator of chromosomal rearrangements.^21^ To date, no studies have been found that the association between RET/PTC1 and NHEJ after IR. Here, we employed three specific inhibitors: SCR7 to inhibit NHEJ, RI‐1 to obstruct HR, and Olaparib to inhibit MMEJ. We observed that the number of RET/PTC1 rearrangement decreased when NHEJ was inhibited by SCR7 after exposure to 0.5 Gy IR, while it increased with RI‐1. No significant alteration in RET/PTC1 rearrangements was detected with Olaparib after 0.5 Gy IR exposure. Thus, our results suggested that not HR or MMEJ but NHEJ plays a crucial role in the formation of RTE/PTC1 rearrangements induced by low dose of IR.

DNA‐PK is recognized as a critical protein kinase in the NHEJ pathway, primarily due to its essential role in the assembly of the holoenzyme complex. This assembly is vital for recruiting NHEJ‐related ligases and repair proteins, such as DNA ligase IV, XRCC4, XRCC4‐like factor, and Artemis.^52^, ^53^ It is generally believed that heterodimers composed of Ku70 and Ku80 first recognize DSBs, subsequently recruiting and activating DNA‐PKcs.^54^, ^55^, ^56^, ^57^ DNA‐PKcs, a serine/threonine kinase, has been demonstrated to phosphorylate various proteins in vitro, such as p53, transcription factors, RNA polymerases, and Ku70/Ku80.^22^, ^58^, ^59^, ^60^ Despite DNA‐PKcs's crucial role in NHEJ, its involvement in IR‐induced chromosome rearrangement in thyroid follicular epithelial cells remains unclear.^22^, ^61^ Our findings revealed that both knockdown of DNA‐PKcs and inhibition of its phosphorylation reduced in NHEJ efficiency, thereby reducing the incidence of RET/PTC1 rearrangements in thyroid follicular epithelial cells exposed to 0.5 Gy IR. In this study, we have only been briefly examined the extensive capabilities of DNA‐PKcs in relation to its function in the NHEJ repair efficiency domain. DNA‐PKcs, within the human body, generates its own protective effects against IR and oxidative stress. For instance, it facilitates the production of superoxide dismutase, enabling organs to endure higher doses of IR compared to isolated cell lines, even when both experience equivalent levels of damage from exposure.^23^, ^62^, ^63^, ^64^, ^65^ Consequently, exposure doses on cell lines and experiments can only serve as a cautionary measure for individuals at risk of IR exposure. Our current study has identified that DNA‐PKcs‐mediated alterations in NHEJ plays a crucial role in the formation of RET/PTC1 rearrangements in thyroid follicular epithelial cells following low‐dose IR exposure.

In summary, we identify that the response of thyroid follicular epithelial cells to low dose of IR can undergo RET/PTC1 rearrangement. Our data emphasize the importance of NHEJ between RET/PTC1 rearrangement and IR, particularly highlighting the involvement of DNA‐PKcs in facilitating RET/PTC1 rearrangement. Consequently, targeting DNA‐PKcs to inhibit the NHEJ pathway has emerged as a promising therapeutic strategy for addressing IR‐induced RET/PTC1 rearrangement in PTC. Despite this, there are three aspects of IR‐induced RET/PTC1 in PTC that warrant further study. First, it is crucial to develop specific protective agents against thyroid exposure to low dose IR, particularly from the perspective of NHEJ. Second, although the RET and CCDC6 genes are located on the same chromosome, they are positioned far apart, which allows for the formation of various RET/PTC rearrangements uniquely in thyroid cells.^44^, ^46^ This raises the question of whether the chromosomal structure of thyroid cells differs fundamentally from that of other cell types, thereby facilitating RET/PTC rearrangement. Third, both high and low doses of IR can lead to RET/PTC1 rearrangement, suggesting potentially distinct underlying mechanisms that need thorough investigation. Research into these areas will enhance our understanding of the molecular processes involved and may lead to better prevention and treatment strategies for PTC.

## 4 MATERIALS AND METHODS

### 4.1 Cell culture

The human normal thyroid epithelial cell line (N‐thy‐ori‐3‐1) and thyroid papillary carcinoma cell line (TPC‐1: CVCL_6298), purchased from the Pricella Life Science & Technology, were grown in Roswell Park Memorial Institute 1640 (RPMI 1640 Sigma, Inc.), supplemented with 1% antibiotics/antimycotics (Invitrogen) and 10% fetal bovine serum (FBS) (ExCell Bio). The HLF cell line (Human Lung Fibroblasts) purchased from Pricella Life Science & Technology, was cultured in Dulbecco's Modified Eagle Medium (DMEM, Sigma, Inc.) supplemented with 10% FBS (ExCell Bio) and 1% antibiotics/antimycotics (Invitrogen).

### 4.2 Cell irradiation

Trypsin digestion of well‐grown N‐thy‐ori‐3‐1 cells was carried out and counted to ensure that the number of cells per well in a six‐well plate was 2 × 10^4^ cells. After that, the cells were passaged into six‐well plates for 16 h and then exposed with a single dose of IR from a γ ^60^Co source at a dose rate of 60 cGy/min. Cobalt‐60 (^60^Co), an artificial radionuclide that emits high‐energy electrons, is a commonly acceptable source of gamma‐ray radiation. Cells were cultured in a humidified thermostatic incubator at 37°C containing 5% CO_2_, according to the experimental design, following grown for 3−6 days and harvested.

### 4.3 Gradient dilution experiment

To assess the sensitivity of the nested PCR for the detection of RET/PTC1, and to determine the number of cells to be inoculated and the limits of the assay for each subsequent independent experiment. A gradient dilution experiment was performed by mixing 10^6^, 10^5^, 10^4^,10^3^, 10^2^, 10^1^, and 10^0^ TPC‐1 cells with 10^6^ thyroid follicular epithelial cells to test the accuracy of the experiment. The sensitivity of the assay was determined to be one positive cell mixed in 10^7^ during this study. To ensure the accuracy of the experiments, 10^6^ cells were used, and irradiation was performed after inoculating them in six‐well plates.

### 4.4 Detection of RET/PTC1 rearrangements

Total RNA was isolated by TRIzol from cells (Ambion, Thermo Fisher Scientific) and eluted in 20 µL of RNase/DNase‐free buffer (Biomed, RA114‐02). A Nanodrop 2000c spectrophotometer (Thermo Fisher Scientific) was employed to determine RNA concentration and quality. RNA reverse transcription was performed with Rever Tra Ace qPCR RT Master Mix and gDNA Removal Kit (Large Edition). The reverse transcription system was carried by Applied Biosystems PCR (Ambion, Thermo Fisher Scientific). The first and the nested PCRs were carried out with 35 cycles in Applied Biosystems PCR (Applied Biosystems). The first‐round PCR products were pooled, diluted 1:5, and then, 1 µL was used for one nested PCR. The PCR amplification products were analyzed with 2%−3% agarose (Beyotime, ST004L) gel electrophoresis and ethidium bromide staining (Thermo Fisher Scientific, 15585011). The specificity of the amplification product with predicted size was confirmed by Sanger sequencing. TPC‐1 cells were used as a positive control and HLF cells were used as a negative control.^40^, ^66^ The primers used are listed in Table S2.^40^, ^67^

### 4.5 Antibodies and chemicals

Anti‐GAPDH (Santa Cruz, sc‐32233), anti‐H2AX (Santa Cruz, sc‐517336), anti‐γH2AX (Santa Cruz, sc‐517348), anti‐CHK1 (Santa Cruz, sc‐84084), anti‐pCHK1 Ser345 (CST, 2348S), anti‐DNA‐PKcs (Invitrogen; Thermo Fisher Scientific, Inc.; MA5‐13238), anti‐pDNA‐PKcs S2056 (Abcam; cat. no. ab18192), anti‐STAT3 (CST, 9139S), anti‐p‐STAT3‐Tyr705 (CST, 9145S), anti‐AKT (CST, 9272S), anti‐pAKT‐Ser473 (CST, 4060S), anti‐ERK1/2 (CST, 9102S), and anti‐p‐ERK1/2‐Thr202/Tyr204 (CST, 9102S). All antibodies were diluted at 1:1000. The chemical Rad51 inhibitor RI‐1 (cat. no. S8077), DNA ligase IV inhibitor SCR7 (cat. no. S7742), DNA‐PK inhibitor Nu7441 (cat. no. S2638), Parp1 inhibitor Olaparib (cat. no. S1060), and thymidine (cat. no. S4803) were purchased from Selleck Chemicals.

### 4.6 Western blotting procedures

At 4°C, cells were washed with phosphate buffered saline(PBS), collected into EP tubes by centrifuging at 3000 rpm for 3 min, lysed on ice for 30 min with RIPA (Beyotime, P0013B), and centrifuged at 12,000 rpm for 10 min. The protein concentration was measured by Nanodrop 2000c spectrophotometer and 40 µg was loaded per lane on 6%−12% SDS–PAGE gels according to molecular weight of proteins. Proteins were transferred to nitrocellulose membranes and blocked with 5% bovine serum albumin(BSA) for 1 h at room temperature. Membranes were incubated overnight at 4°C with primary antibodies. After washing, membranes were incubated with secondary antibodies (1:4000) at room temperature for 1 h. After washing, SuperSignal West Pico PLUS Chemiluminescent Substrate (Thermo Fisher Scientific) was uniformly added to the membrane. The bands were visualized using an Image Quant LAS 500 and the Image Quant LAS 500 1.1.0 software (GE Healthcare Life Sciences).

### 4.7 Transfection

The siRNA and plasmid used in the present study were purchased from Gene Pharma (Gene Pharma); the sequences were as follows: siRET/PTC1.1—5′‐AACTATCAAACGTGTCC‐3′; siRET/PTC1.2—5′‐AACATCTCGGGACAAGC‐3′; siRET/PTC1.3—5′‐TATCTTCAGCACCTTGTTC‐3′; siDPK1—5′‐GGGCGCTAATCGTACTGAA‐3′; and siDPK2—5′‐GGAAGAAGGUGAAGGAGAATT‐3′. All transfections were conducted using lipofectamine 2000 (Invitrogen, 11668019) according to the manufacturer's instructions.

### 4.8 NHEJ assay

NHEJ‐GFP plasmid was digested with HindIII enzyme overnight at 37°C and recovered using AxyPrep DNA Gel Extraction Kit (Axygen; Corning, Inc.) according to the manufacturer's instructions. N‐thy‐ori‐3‐1 cells (3 × 10^5^) were pretreated with SCR7 (50 µM) or RI‐1 (30 µM) for 1 h. The cells were transfected with 0.25 µg of pCherry and 1 µg of the digested NHEJ‐GFP plasmid and mixed with 2.5 µL of Lipofectamine 2000. Following 6 h, the culture medium of the transfected cells was replaced with medium containing SCR7 (50 µM) or RI‐1 (30 µM) and further cultured for 2 days. The detailed procedure has been described.^68^ The cells were trypsinized (0.25%) and resuspended in PBS. The cellular fluorescence was measured by flow cytometry analysis (NovoCyte; ACEA Biosciences, Inc.) and the NovoExpress 1.3.0 software (ACEA Biosciences, Inc.).

### 4.9 HR assay

N‐thy‐ori‐3‐1 cells (3 × 10^5^) were pretreated with SCR7 (50 µM) or RI‐1 (30 µM) for 1 h. Then, they were transfected with a single copy of a DR‐GFP, I‐SceI expression plasmid and with a pCherry plasmid used as a transfection efficiency control. Following 6 h, the culture medium of the transfected cells was replaced with medium containing SCR7 (50 µM) or RI‐1 (30 µM), after which the cells were further cultured for 2 days and subjected to flow cytometry analysis (NovoCyte; ACEA Biosciences, Inc.) and the NovoExpress 1.3.0 software (ACEA Biosciences, Inc.), and then, the GFP‐positive cell population was measured. The mean values were obtained from three independent experiments. Little variation was observed among the three independent experiments.^68^

### 4.10 Real‐time PCR assay

Total cellular RNA was extracted by TRIzol and the reverse transcription procedure was carried out according to the product instructions. The primers were designed and synthesized by Tingke Biotech. β‐Actin was used for normalization as a control.

### 4.11 Animal experiments

BALB/c‐nu mice (4 weeks old) were purchased from SPF (Beijing) Biotechnology Co., Ltd., and experiment was conducted after 1 week of stabilization. TPC‐1 (siRET/PTC1) and N‐thy‐ori‐3‐1 (OE RET/PTC1) cells were digested down separately with trypsin, washed twice with PBS, and 0.2 mL cell suspension was taken at a concentration of 3 × 10^7^/mL and injected into the right subcutaneous side of nude mice. The animals were housed in ventilated microisolators with free access to sterile food and water. Twenty‐one days after injection, BALB/c‐nu mice were sacrificed, and tumors were observed, collected, weighted, and measured.

### 4.12 Cell proliferation assay

For CCK‐8 assay, cells were diluted with complete medium to a concentration of 15,000/mL and added to 96‐well plates for culture at 100 µL per well. Absorbance values were measured at 12/24/48/72 h after cell inoculation. For the colony formation assay, cells were diluted with complete medium to a concentration of 1000/mL and added to six‐well plates for culture. After 10 days of culture, the cell colonies were fixed with 4% paraformaldehyde (Beyotime, P0099) and stained with Giemsa (Beyotime, C0133).

### 4.13 Cell cycle synchronization using thymidine double block

The cells were initially cultured to approximately 50%‒60% confluence in complete growth medium. The first block was achieved by incubating cells with 2 mM thymidine for 16 h. Following this, cells were released from the block by washing three times with PBS and incubating them in fresh thymidine‐free medium for 8−10 h to allow cell cycle progression. A second thymidine block was then applied by re‐treating the cells with 2 mM thymidine for an additional 16 h. After the second block, cells were washed thoroughly with PBS to remove residual thymidine and incubated in fresh medium. Synchronization efficiency was assessed by flow cytometry.

### 4.14 Statistical analysis

Statistical analyses were conducted with GraphPad Prism (version 8.0) software. The data were derived from at least three independent experiments and are presented as mean ± SEM. *p *< 0.05 was considered statistically significant.

## AUTHOR CONTRIBUTIONS

Yuhao Liu and Jiaojiao Zhu conducted the experiments, performed data analysis, and drafted the manuscript. Shenghui Zhou conducted the experiments. Yifan Hou, Ziyan Yan, Xingkun Ao, Ping Wang, and Lin Zhou provided experimental support. Huixi Chen, Xinxin Liang, Hua Guan, Shanshan Gao, and Dafei Xie provided experimental reagents and materials. Yongqing Gu and Ping‐Kun Zhou supervised the project, and designed, edited, and led out the experiments of this study. All authors have read and approved the final manuscript.

## CONFLICT OF INTEREST STATEMENT

The authors declare they have no conflicts of interest.

## ETHICS STATEMENT

All the animal experiments were performed in compliance with the Guide for the Care and Use of Laboratory Animals and were performed according to the guidelines for the Laboratory Animal Guideline of Welfare and Ethics of China. All experimental procedures were approved by the Animal Care and Use Committee at the Military Academy of Medical Sciences. (IACUC‐DWZX‐2024‐531).

## Supporting information

Supporting Information

## Data Availability

The data that support the findings of this study are available from the corresponding author upon reasonable request.

## References

[1] Albi E , Cataldi S , Lazzarini A , et al. Radiation and thyroid cancer. Int J Mol Sci. 2017;18(5):911. 28445397 10.3390/ijms18050911 PMC5454824

[2] Cabanillas ME , McFadden DG , Durante C . Thyroid cancer. Lancet. 2016;388(10061):2783‐2795. 27240885 10.1016/S0140-6736(16)30172-6

[3] Carling T , Udelsman R . Thyroid cancer. Annu Rev Med. 2014;65:125‐137. 24274180 10.1146/annurev-med-061512-105739

[4] Virginia AL . Papillary thyroid carcinoma: an update. Mod Pathol. 2011;24(suppl 2):S1‐S9. 21455196 10.1038/modpathol.2010.129

[5] Pacini F , Vorontsova T , Demidchik EP , et al. Post‐Chernobyl thyroid carcinoma in Belarus children and adolescents: comparison with naturally occurring thyroid carcinoma in Italy and France. J Clin Endocrinol Metab. 1997;82(11):4367. 10.1210/jcem.82.11.4367 9360507

[6] Elisei R , Romei C , Vorontsova T , et al. RET/PTC rearrangements in thyroid nodules: studies in irradiated and not irradiated, malignant and benign thyroid lesions in children and adults. J Clin Endocrinol Metab. 2001;86(7):3211‐3216. 11443191 10.1210/jcem.86.7.7678

[7] Robbins J , Schneider AB . Thyroid cancer following exposure to radioactive iodine. Rev Endocr Metab Disord. 2000;1(3):197‐203. 11705004 10.1023/a:1010031115233

[8] Ricarte‐Filho JC , Li S , Garcia‐Rendueles ME , et al. Identification of kinase fusion oncogenes in post‐Chernobyl radiation‐induced thyroid cancers. J Clin Invest. 2013;123(11):4935‐4944. 24135138 10.1172/JCI69766 PMC3809792

[9] Ashwini BR , Nirmala C , Natarajan M , Biligi DS . A study to evaluate association of nuclear grooving in benign thyroid lesions with RET/PTC1 and RET/PTC3 gene translocation. Thyroid Res. 2023;16(1):21. 37394464 10.1186/s13044-023-00161-9 PMC10316543

[10] Nikiforov YE . RET/PTC rearrangement in thyroid tumors. Endocr Pathol. 2002;13(1):3‐16. 12114746 10.1385/ep:13:1:03

[11] Tallini G , Asa SL . RET oncogene activation in papillary thyroid carcinoma. Adv Anat Pathol. 2001;8(6):345‐354. 11707626 10.1097/00125480-200111000-00005

[12] Burssed B , Zamariolli M , Bellucco FT , Melaragno MI . Mechanisms of structural chromosomal rearrangement formation. Mol Cytogenet. 2022;15(1):23. 35701783 10.1186/s13039-022-00600-6 PMC9199198

[13] Carvalho CM , Lupski JR . Mechanisms underlying structural variant formation in genomic disorders. Nat Rev Genet. 2016;17(4):224‐238. 26924765 10.1038/nrg.2015.25 PMC4827625

[14] Caudill CM , Zhu Z , Ciampi R , Stringer JR , Nikiforov YE . Dose‐dependent generation of RET/PTC in human thyroid cells after in vitro exposure to gamma‐radiation: a model of carcinogenic chromosomal rearrangement induced by ionizing radiation. J Clin Endocrinol Metab. 2005;90(4):2364‐2369. 15671095 10.1210/jc.2004-1811

[15] Ottaviani D , LeCain M , Sheer D . The role of microhomology in genomic structural variation. Trends Genet. 2014;30(3):85‐94. 24503142 10.1016/j.tig.2014.01.001

[16] Hattori A , Fukami M . Established and novel mechanisms leading to de novo genomic rearrangements in the human germline. Cytogenet Genome Res. 2020;160(4):167‐176. 32396893 10.1159/000507837

[17] Wray J , Williamson EA , Singh SB , et al. PARP1 is required for chromosomal translocations. Blood. 2013;121(21):4359‐4365. 23568489 10.1182/blood-2012-10-460527 PMC3663429

[18] Soni A , Siemann M , Grabos M , Murmann T , Pantelias GE , Iliakis G . Requirement for Parp‐1 and DNA ligases 1 or 3 but not of Xrcc1 in chromosomal translocation formation by backup end joining. Nucleic Acids Res. 2014;42(10):6380‐6392. 24748665 10.1093/nar/gku298 PMC4041464

[19] Ahrabi S , Sarkar S , Pfister SX , et al. A role for human homologous recombination factors in suppressing microhomology‐mediated end joining. Nucleic Acids Res. 2016;44(12):5743‐5757. 27131361 10.1093/nar/gkw326 PMC4937322

[20] Gu W , Zhang F , Lupski JR . Mechanisms for human genomic rearrangements. PathoGenetics. 2008;1(1):4. 19014668 10.1186/1755-8417-1-4 PMC2583991

[21] Huang RX , Zhou PK . DNA damage response signaling pathways and targets for radiotherapy sensitization in cancer. Signal Transduct Target Ther. 2020;5(1):60. 32355263 10.1038/s41392-020-0150-x PMC7192953

[22] Fang X , Huang Z , Zhai K , et al. Inhibiting DNA‐PK induces glioma stem cell differentiation and sensitizes glioblastoma to radiation in mice. Sci Transl Med. 2021;13(600):eabc7275. 34193614 10.1126/scitranslmed.abc7275

[23] Gerlach BD , Ampomah PB , Yurdagul A Jr , et al. Efferocytosis induces macrophage proliferation to help resolve tissue injury. Cell Metab. 2021;33(12):2445‐2463. e8. 34784501 10.1016/j.cmet.2021.10.015 PMC8665147

[24] Gao SS , Guan H , Yan S , et al. TIP60 K430 SUMOylation attenuates its interaction with DNA‐PKcs in S‐phase cells: facilitating homologous recombination and emerging target for cancer therapy. Sci Adv. 2020;6(28):eaba7822. 32832608 10.1126/sciadv.aba7822 PMC7439314

[25] Xie Y , Liu YK , Guo ZP , et al. RBX1 prompts degradation of EXO1 to limit the homologous recombination pathway of DNA double‐strand break repair in G1 phase. Cell Death Differ. 2020;27(4):1383‐1397. 31562368 10.1038/s41418-019-0424-4 PMC7205894

[26] van Gerwen M , Alerte E , Alsen M , Little C , Sinclair C , Genden E . The role of heavy metals in thyroid cancer: a meta‐analysis. J Trace Elem Med Biol. 2021;69:126900. 34798515 10.1016/j.jtemb.2021.126900

[27] Shkala K , Zhenyu Z , Whitney S , et al. Ambient particulate matter air pollution is associated with increased risk of papillary thyroid cancer. Surgery. 2021;171(1):212‐219. 34210530 10.1016/j.surg.2021.05.002 PMC8688174

[28] Gore AC , Chappell VA , Fenton SE , et al. EDC‐2: the endocrine society's second scientific statement on endocrine‐disrupting chemicals. Endocr Rev. 2015;36(6):E1‐E150. 26544531 10.1210/er.2015-1010 PMC4702494

[29] Yu‐Jin K , Hye‐Sun L , Sang‐Wook K , Ji‐Won L . Association between consumption of iodine‐rich foods and thyroid cancer prevalence: findings from a large population‐based study. Nutrients. 2024;16(7):1041. 38613074 10.3390/nu16071041 PMC11013877

[30] Hoskins SB , Torgerson L . Synchronous papillary thyroid cancer and colorectal cancer in a young patient with a CHEK2 mutation. Case Rep Oncol. 2024;17(1):524‐531. 38567167 10.1159/000536052 PMC10987181

[31] Richardson DB . Exposure to ionizing radiation in adulthood and thyroid cancer incidence. Epidemiology. 2009;20(2):181‐187. 19177023 10.1097/EDE.0b013e318196ac1c

[32] Kitahara CM , Preston DL , Neta G , et al. Occupational radiation exposure and thyroid cancer incidence in a cohort of U.S. radiologic technologists, 1983–2013. Int J Cancer. 2018;143(9):2145‐2149. 29355960 10.1002/ijc.31270 PMC6054904

[33] Lin Y , Wu Y . Trends in incidence and overdiagnosis of thyroid cancer in China, Japan, and South Korea. Cancer Sci. 2023;114(10):4052‐4062. 37488752 10.1111/cas.15909 PMC10551580

[34] Li M , Zheng R , Dal Maso L , Zhang S , Wei W , Vaccarella S . Mapping overdiagnosis of thyroid cancer in China. Lancet Diabetes Endocrinol. 2021;9(6):330‐332. 33891886 10.1016/S2213-8587(21)00083-8

[35] Miranda‐Filho A , Lortet‐Tieulent J , Bray F , et al. Thyroid cancer incidence trends by histology in 25 countries: a population‐based study. Lancet Diabetes Endocrinol. 2021;9(4):225‐234. 33662333 10.1016/S2213-8587(21)00027-9

[36] Mizuno T , Iwamoto KS , Kyoizumi S , et al. Preferential induction of RET/PTC1 rearrangement by X‐ray irradiation. Oncogene. 2000;19(3):438‐443. 10656692 10.1038/sj.onc.1203343

[37] Barone MV , Sepe L , Melillo RM , et al. RET/PTC1 oncogene signaling in PC Cl 3 thyroid cells requires the small GTP‐binding protein Rho. Oncogene. 2001;20(48):6973‐6982. 11704822 10.1038/sj.onc.1204886

[38] Gilbert‐Sirieix M , Ripoche H , Malvy C , Massaad‐Massade L . Effects of silencing RET/PTC1 junction oncogene in human papillary thyroid carcinoma cells. Thyroid. 2010;20(10):1053‐1065. 20615140 10.1089/thy.2010.0006

[39] Cassinelli G , Favini E , Degl'Innocenti D , et al. RET/PTC1‐driven neoplastic transformation and proinvasive phenotype of human thyrocytes involve Met induction and beta‐catenin nuclear translocation. Neoplasia. 2009;11(1):10‐21. 19107227 10.1593/neo.08916 PMC2606114

[40] Ameziane‐El‐Hassani R , Boufraqech M , Lagente‐Chevallier O , et al. Role of H_2_O_2_ in RET/PTC1 chromosomal rearrangement produced by ionizing radiation in human thyroid cells. Cancer Res. 2010;70(10):4123‐4132. 20424115 10.1158/0008-5472.CAN-09-4336

[41] Mitsutake N , Saenko V . Molecular pathogenesis of pediatric thyroid carcinoma. J Radiat Res. 2021;62:i71‐i77. 33978172 10.1093/jrr/rraa096 PMC8114219

[42] Allocca C , Cirafici A , Laukkanen M , Castellone M . Serine 897 phosphorylation of EPHA2 is involved in signaling of oncogenic ERK1/2 drivers in thyroid cancer cells. Thyroid. 2021;31(1):76‐87. 32762307 10.1089/thy.2019.0728

[43] Lehman C , Dillon L , Nikiforov Y , Wang Y . DNA fragile site breakage as a measure of chemical exposure and predictor of individual susceptibility to form oncogenic rearrangements. Carcinogenesis. 2017;38(3):293‐301. 28069693 10.1093/carcin/bgw210 PMC5862292

[44] Nikiforova MN , Stringer JR , Blough R , Medvedovic M , Fagin JA , Nikiforov YE . Proximity of chromosomal loci that participate in radiation‐induced rearrangements in human cells. Science. 2000;290(5489):138‐141. 11021799 10.1126/science.290.5489.138

[45] Celetti A , Cerrato A , Merolla F , Vitagliano D , Vecchio G , Grieco M . H4(D10S170), a gene frequently rearranged with RET in papillary thyroid carcinomas: functional characterization. Oncogene. 2004;23(1):109‐121. 14712216 10.1038/sj.onc.1206981

[46] Gandhi M , Medvedovic M , Stringer JR , Nikiforov YE . Interphase chromosome folding determines spatial proximity of genes participating in carcinogenic RET/PTC rearrangements. Oncogene. 2006;25(16):2360‐2366. 16331264 10.1038/sj.onc.1209268

[47] Jackson SP , Bartek J . The DNA‐damage response in human biology and disease. Nature. 2009;461(7267):1071‐1078. 19847258 10.1038/nature08467 PMC2906700

[48] Shimizu I , Yoshida Y , Suda M , Minamino T . DNA damage response and metabolic disease. Cell Metab. 2014;20(6):967‐977. 25456739 10.1016/j.cmet.2014.10.008

[49] Ciccia A , Elledge SJ . The DNA damage response: making it safe to play with knives. Mol Cell. 2010;40(2):179‐204. 20965415 10.1016/j.molcel.2010.09.019 PMC2988877

[50] Chang HHY , Pannunzio NR , Adachi N , Lieber MR . Non‐homologous DNA end joining and alternative pathways to double‐strand break repair. Nat Rev Mol Cell Biol. 2017;18(8):495‐506. 28512351 10.1038/nrm.2017.48 PMC7062608

[51] Zhao B , Rothenberg E , Ramsden DA , Lieber MR . The molecular basis and disease relevance of non‐homologous DNA end joining. Nat Rev Mol Cell Biol. 2020;21(12):765‐781. 33077885 10.1038/s41580-020-00297-8 PMC8063501

[52] Yue X , Bai C , Xie D , Ma T , Zhou PK . DNA‐PKcs: a multi‐faceted player in DNA damage response. Front Genet. 2020;11:607428. 33424929 10.3389/fgene.2020.607428 PMC7786053

[53] Chen X , Xu X , Chen Y , et al. Structure of an activated DNA‐PK and its implications for NHEJ. Mol Cell. 2021;81(4):801‐810. e3. 33385326 10.1016/j.molcel.2020.12.015 PMC7897279

[54] Gottlieb TM , Jackson SP . The DNA‐dependent protein kinase: requirement for DNA ends and association with Ku antigen. Cell. 1993;72(1):131‐142. 8422676 10.1016/0092-8674(93)90057-w

[55] Hartley KO , Gell D , Smith GC , et al. DNA‐dependent protein kinase catalytic subunit: a relative of phosphatidylinositol 3‐kinase and the ataxia telangiectasia gene product. Cell. 1995;82(5):849‐856. 7671312 10.1016/0092-8674(95)90482-4

[56] Baumann P , West SC . DNA end‐joining catalyzed by human cell‐free extracts. Proc Nat Acad Sci U S A. 1998;95(24):14066‐14070. 10.1073/pnas.95.24.14066 PMC24327 9826654

[57] Jackson SP , Jeggo PA . DNA double‐strand break repair and V(D)J recombination: involvement of DNA‐PK. Trends Biochem Sci. 1995;20(10):412‐415. 8533154 10.1016/s0968-0004(00)89090-8

[58] Zhou H , Du W , Li Y , et al. Effects of melatonin on fatty liver disease: the role of NR4A1/DNA‐PKcs/p53 pathway, mitochondrial fission, and mitophagy. J Pineal Res. 2018;64(1):e12450. 10.1111/jpi.12450 28981157

[59] Sirbu BM , Cortez D . DNA damage response: three levels of DNA repair regulation. Cold Spring Harb Perspect Biol. 2013;5(8):a012724. 23813586 10.1101/cshperspect.a012724 PMC3721278

[60] Iliakis GE . New players in the regulation of DNA‐PK activity: survivin joins the crowd. Cancer Res. 2021;81(9):2270‐2271. 34003784 10.1158/0008-5472.CAN-21-0273

[61] Zhao Y , Thomas HD , Batey MA , et al. Preclinical evaluation of a potent novel DNA‐dependent protein kinase inhibitor NU7441. Cancer Res. 2006;66(10):5354‐5362. 16707462 10.1158/0008-5472.CAN-05-4275

[62] Han Y , Jin F , Xie Y , et al. DNA‑PKcs PARylation regulates DNA‑PK kinase activity in the DNA damage response. Mol Med Rep. 2019;20(4):3609‐3616. 31485633 10.3892/mmr.2019.10640 PMC6755157

[63] Guo Z , Wang S , Xie Y , et al. HUWE1‐dependent DNA‐PKcs neddylation modulates its autophosphorylation in DNA damage response. Cell Death Dis. 2020;11(5):400. 32457294 10.1038/s41419-020-2611-0 PMC7250858

[64] Chen Y , Jiang T , Zhang H , et al. LRRC31 inhibits DNA repair and sensitizes breast cancer brain metastasis to radiation therapy. Nat Cell Biol. 2020;22(10):1276‐1285. 33005030 10.1038/s41556-020-00586-6 PMC7962994

[65] Holley A , Xu Y , St Clair D , St Clair W . RelB regulates manganese superoxide dismutase gene and resistance to ionizing radiation of prostate cancer cells. Ann NY Acad Sci. 2010;1201:129‐136. 20649549 10.1111/j.1749-6632.2010.05613.x PMC3107504

[66] Unger K , Zurnadzhy L , Walch A , et al. RET rearrangements in post‐Chernobyl papillary thyroid carcinomas with a short latency analysed by interphase FISH. Br J Cancer. 2006;94(10):1472‐1477. 16641909 10.1038/sj.bjc.6603109 PMC2365029

[67] Bounacer A , Wicker R , Caillou B , et al. High prevalence of activating ret proto‐oncogene rearrangements, in thyroid tumors from patients who had received external radiation. Oncogene. 1997;15(11):1263‐1273. 9315093 10.1038/sj.onc.1200206

[68] Seluanov A , Mao Z , Gorbunova V . Analysis of DNA double‐strand break (DSB) repair in mammalian cells. J Vis Exp. 2010;43:2002. 10.3791/2002 PMC3157866 20864925

