# Supplementary material for: Low‐dose ionizing radiation‐induced RET/PTC1 rearrangement via the non‐homologous end joining pathway to drive thyroid cancer
Source: MedComm (2020). 2024 Aug 12;5(8):e690. doi: 10.1002/mco2.690 (PMC11318340; doi:10.1002/mco2.690)
Supplement: Supplementary file 1 — Supporting Information [file MCO2-5-e690-s001.pdf]

Low dose ionizing radiation-induced RET/PTC1 rearrangement via the non-homologous end joining pathway to drive thyroid cancer

Short title: LDR induced RET/PTC1 rearrangement drives PTC

Yuhao Liu[1#], Jiaojiao Zhu[1#], Shenghui Zhou[2#], Yifan Hou[3], Ziyan Yan[1], Xingkun Ao[2], Ping Wang[1], Lin Zhou[1], Huixi Chen[2], Xinxin Liang[2], Hua Guan[1], Shanshan Gao[1], Dafei Xie[1], Yongqing Gu[1,2,3*] and Ping-Kun Zhou[1*]

[1]Beijing Key Laboratory for Radiobiology, Beijing Institute of Radiation Medicine, Beijing 100850, China

[2]Hengyang Medical College, University of South China, Hengyang, Hunan 421001, China

[3]College of Life Sciences, Hebei University, Baoding 071001, China

*Corresponding authors: Yongqing Gu, Beijing Key Laboratory for Radiobiology, Beijing Institute of Radiation Medicine, Beijing 100850, China. E-mail: yqgu96@163.com; Ping-Kun Zhou, Beijing Key Laboratory for Radiobiology, Beijing Institute of Radiation Medicine, Beijing 100850, China. E-mail: zhoupk@nic.bmi.ac.cn.

[#] Yuhao Liu, Jiaojiao Zhu and Shenghui Zhou contributed equally to this work

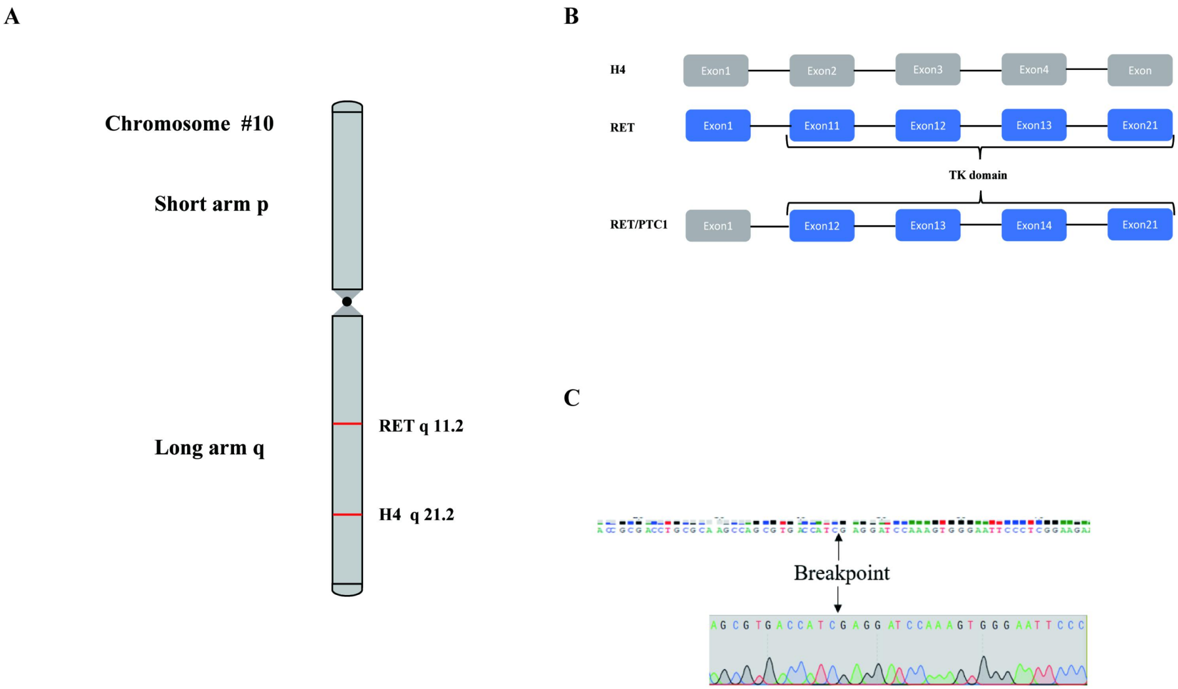

Supplementary Figure 1. RET/PTC1 information on chromosomes. (A) Schematic representation of the location of RET/PTC1 on chromosome; (B) Exon distribution of fusion genes; (C) Samples of normal cells that appeared positive for RET/PTC1 after IR were subjected to Sanger sequencing, which detected the presence of gene breakpoints.

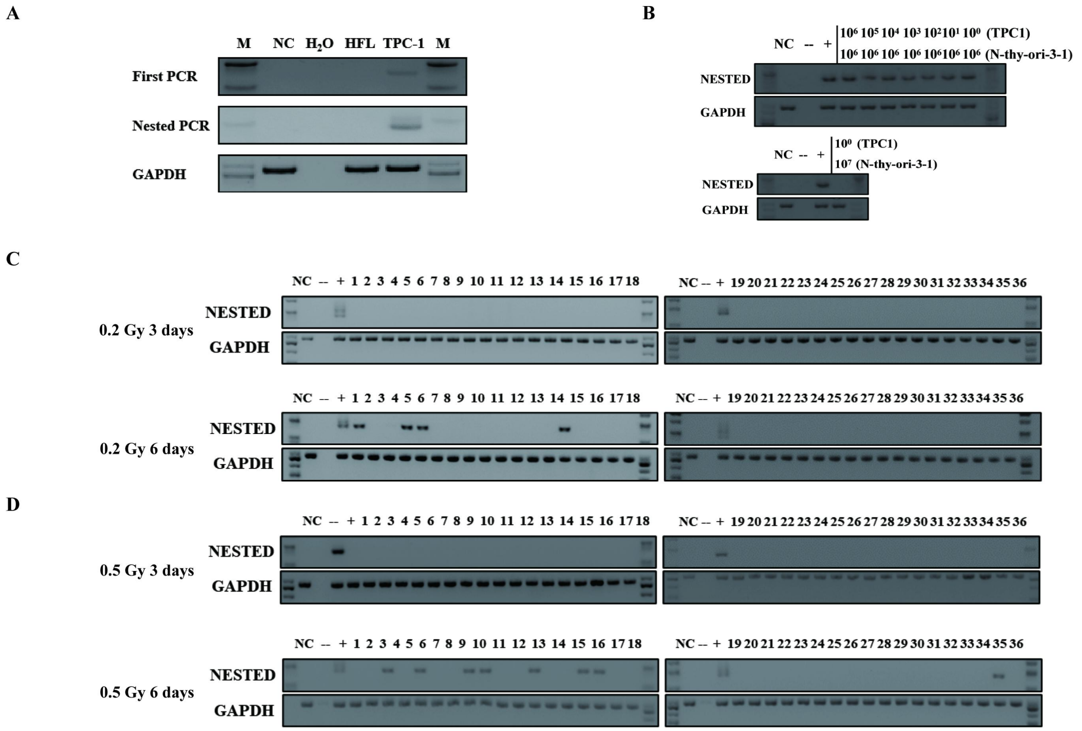

Supplementary Figure 2. Detection of RET/PTC1 in human thyroid epithelial cell. (A) Negative control and positive control about detection of RET/PTC1; (B) Gradient dilution to determine the sensitivity of nest PCR on detection of RET/PTC1; (C) Detection of RET/PTC1 after 0.2 Gy IR at three days and six days; (D) Detection of RET/PTC1 after 0.5 Gy IR at three days and six days. Lane numbers of 1 to 36 correspond with independent cell wells.

A

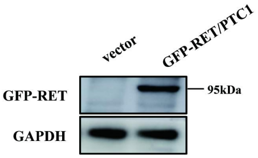

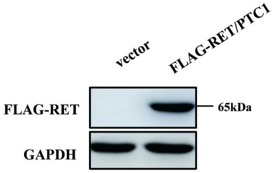

B

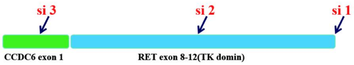

C

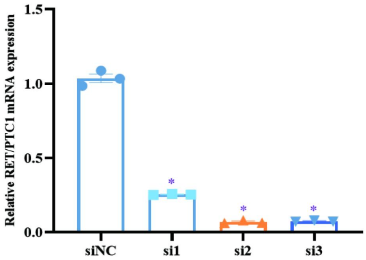

Supplementary Figure 3. Knockdown and overexpression efficiency of RET/PTC1. (A) Overexpression of the RET/PTC1 fusion gene separately with FLAG and GFP tag in normal thyroid follicular epithelial cells; (B) The position of siRNA corresponds to the RET/PTC1 fusion gene; (C) Validation of siRNA knockdown efficiency in TPC-1 cells. Data were derived from at least three independent experiments. Data are shown as means ± SEM. Comparison by unpaired t test, $^*p$<0.05.

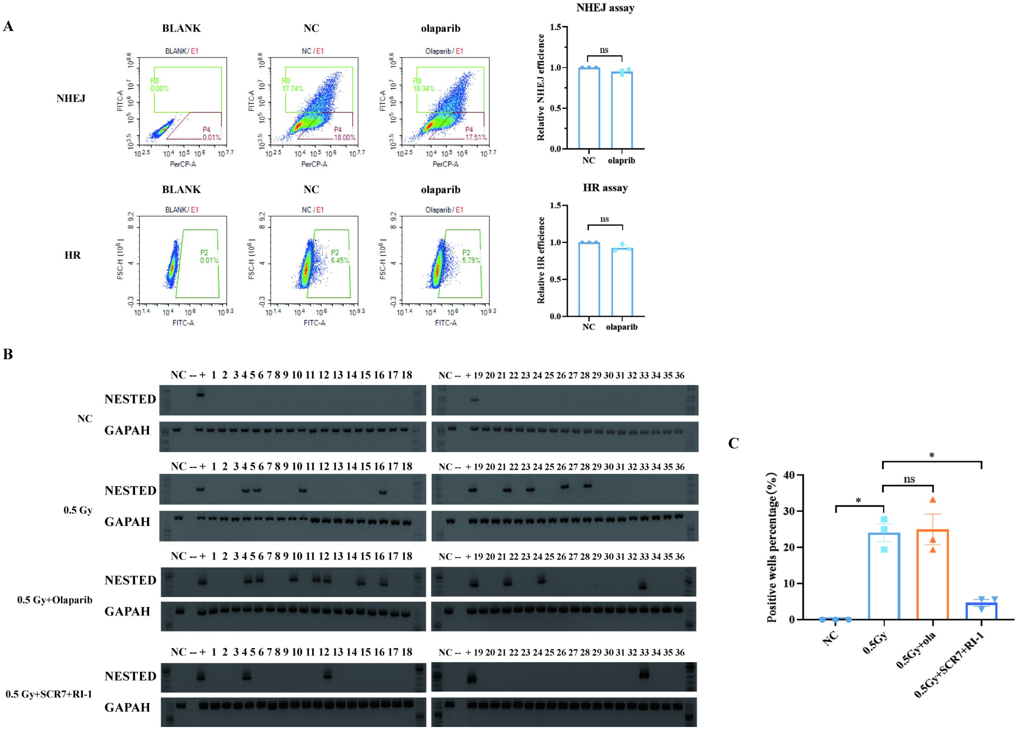

Supplementary Figure 4. Effect of olaparib or concomitant use of NHEJ and HR inhibitors on RET/PTC1 production after IR. (A) Effect on NHEJ and HR repair efficiency after treating with Olaparib; (B) Detection of RET/PTC1 in N-thy-ori-3-1 cells at six days with different treatments: non-irradiation N-thy-ori-3-1 cells (NC group), 0.5 Gy, 0.5 Gy combining with Olaparib and 0.5 Gy combining with SCR7 and RI-1; (C) Quantitative results of (B). Lane numbers of 1 to 36 correspond with independent cell wells. Data were derived from at least three independent experiments.Data are shown as means ± SEM. Comparison by unpaired t test, $^{*}p<0.05$.

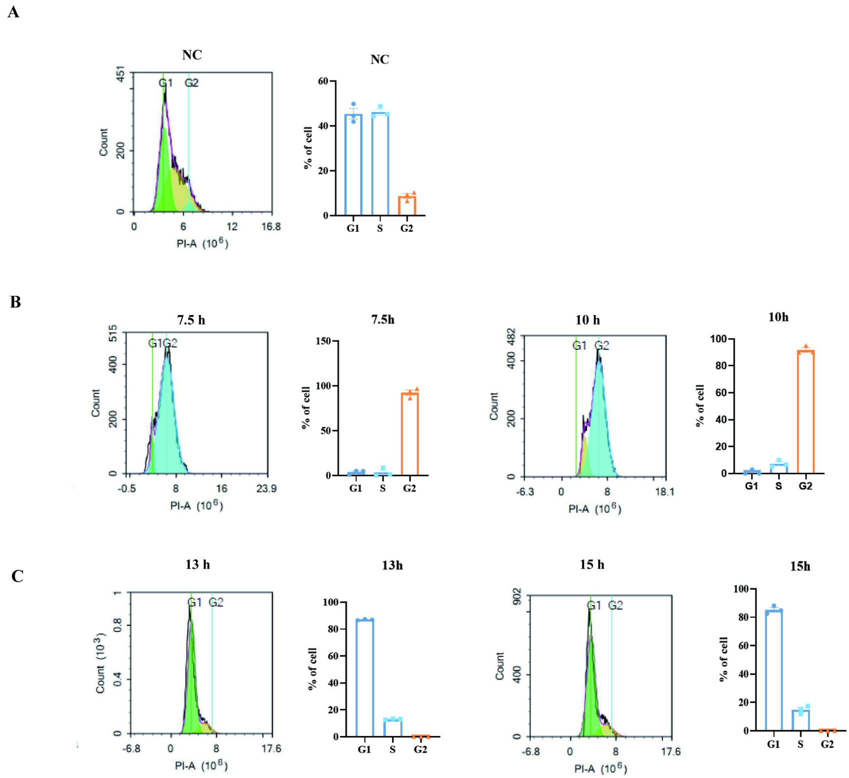

Supplementary Figure 5. Cell cycle synchronization using thymidine double-block by flow cytometry.
(A) Non-treated group; (B) G2-phase cells were collected from 7.5 h to 10 h after releasing from thymidine double-block; (C) G1-phase cells were collected from 13 h to 15 h after releasing from thymidine double-block. Data were derived from at least three independent experiments. Data are shown as means ± SEM. Comparison by unpaired t test, $^{*}p<0.05$.

Supplementary Table 1. There independent experiments of detecting RET/PTC1 with different treatments: non-irradiation Nthy-ori-3-1 cells (NC group), 0.5 Gy, 0.5 Gy combining with Olaparib, and 0.5 Gy combining with SCR7 and RI-1

	Treatments	Wells tested	Wells RET/PTC1 Positives	RET/PTC1(%)
**Experiment1**	Control	36	0	0
	0. 5 Gy	36	8	22.2
	0.5 Gy+Olaparib	36	12	33.3
	0.5 Gy+SCR7+RI-1	36	2	5.6
**Experiment2**	Control	36	0	0
	0.05 Gy	36	8	22.2
	0.5 Gy+ Olaparib	36	8	22.2
	0.5 Gy+ SCR7+RI-1	36	1	2.8
**Experiment3**	Control	36	0	0
	0.05 Gy	36	9	25
	0.5 Gy+ Olaparib	36	7	19.4
	0.5 Gy+ SCR7+RI-1	36	2	5.6

Supplementary Table 2. The primers used in this study for PCR assays

Gene	Primer sequence
GAPDH F	ACCACAGTCCATGCCATCAC
GAPDH R	TCCACCACCCTGTTGCTGTA
RET1 1F	ATTGTCATCTCGCCGTTC
RET1 1R	CTTTCAGCATCTTCACGG
RET1 2F	GCTGGAGACCTACAAACTGA
RET1 2R	CGTTGCCTTGACCACTTTTC
RET/PTC1 F	CCTACAAACTGAAGTGCAAGGC
RET/PTC1 R	CGTGGTGTACCCTGCTCTG
β action F	GACCTGTACGCCAACACAG
β action R	CTCAGGAGGAGCAATGATC
